# Literary Notes

**Published:** 1904-01

**Authors:** 


### LITERARY NOTES.

THE Grafton Press, New York, has entered the medical publishing field and will soon issue a treatise on the surgery of the heart and lungs, by Benjamin Merrill Ricketts, M. D., of Cincinnati. The book will contain about 600 pages of standard octavo text and will be profusely illustrated in an artistic manner. It is announced to issue with the beginning of the new year. Those familiar with the work of the Grafton Press will readily appreciate the high class of printing that characterises all its publications, and this one will be no exception.

---

PRACTICAL *Medicine* is the name of a monthly medical journal, published at Delhi, India, edited by Ram Narain, L. M. S., that appeared during the year 1903. It is a double-column small quarto and contains a variety of articles and items of medical interest. Its advertising pages carry a number of notices of American medical journals.

---

THE *Los Angeles Medical Journal* is a new offering from the Pacific coast that presented itself under date of November 15, 1903. It is edited by Ernest S. Pillsbury, M. D., with a staff of associates. The first number contains 62 pages of medical material, most of which has been judiciously chosen and the journal presents a creditable appearance.

